# Studying Lipid-Related Pathophysiology Using the Yeast Model

**DOI:** 10.3389/fphys.2021.768411

**Published:** 2021-10-28

**Authors:** Tyler Ralph-Epps, Chisom J. Onu, Linh Vo, Michael W. Schmidtke, Anh Le, Miriam L. Greenberg

**Affiliations:** ^1^Department of Biological Sciences, Wayne State University, Detroit, MI, United States; ^2^Muskegon Catholic Central High School, Muskegon, MI, United States

**Keywords:** *Saccharomyces cerevisiae*, lipids, cardiolipin, Barth syndrome, pathophysiology, tafazzin

## Abstract

*Saccharomyces cerevisiae*, commonly known as baker’s yeast, is one of the most comprehensively studied model organisms in science. Yeast has been used to study a wide variety of human diseases, and the yeast model system has proved to be an especially amenable tool for the study of lipids and lipid-related pathophysiologies, a topic that has gained considerable attention in recent years. This review focuses on how yeast has contributed to our understanding of the mitochondrial phospholipid cardiolipin (CL) and its role in Barth syndrome (BTHS), a genetic disorder characterized by partial or complete loss of function of the CL remodeling enzyme tafazzin. Defective tafazzin causes perturbation of CL metabolism, resulting in many downstream cellular consequences and clinical pathologies that are discussed herein. The influence of yeast research in the lipid-related pathophysiologies of Alzheimer’s and Parkinson’s diseases is also summarized.

## Introduction

*Saccharomyces cerevisiae* is a powerful model system used to study biological processes and human diseases. In addition to investigating the pathophysiology of diseases, yeast is also used as a model for developing and testing potential treatments. An excellent example of the power of the yeast model is the use of yeast cardiolipin (CL) synthesis mutants to understand the metabolic abnormalities in Barth syndrome (BTHS), a rare genetic disorder caused by mutations in the CL-remodeling enzyme tafazzin (Bione et al., 1996; Vreken et al., 2000). CL is a unique phospholipid localized primarily in the inner mitochondrial membrane (IMM). Yeast CL mutants have been pivotal in elucidating the role of this lipid in bioenergetics (Paradies et al., 2014; Ren et al., 2014; Raja et al., 2017a), mitochondrial metabolism (Houtkooper and Vaz, 2008; Raja et al., 2019), and programmed cell death (Manon, 2004; Eisenberg and Buttner, 2014; Lou et al., 2018b) among other cellular functions. This review aims to demonstrate how the yeast model has led the way for BTHS studies and contributed to recent advances in our understanding of other human diseases.

## The Mitochondrial Disorder Barth Syndrome

### History

In 1983, physician Peter Barth reported the first description of the disorder that bears his name, describing it as “an X-linked mitochondrial disease affecting cardiac muscle, skeletal muscle, and neutrophil leukocytes” (Barth et al., 1983). It was not until 1996 that the cause of BTHS was linked to mutations in the TAZ gene (Bione et al., 1996). Vreken et al. (2000) discovered that TAZ mutations lead to a profound defect in CL remodeling. During 2003–2004, the first research model for studying BTHS – yeast *taz1*Δ – was constructed (Vaz et al., 2003; Gu et al., 2004; Ma et al., 2004).

Since the emergence of the yeast *taz1*Δ model, numerous other BTHS model systems have been developed in different organisms through targeted disruption of the tafazzin gene. In 2006, the first whole-animal models were generated in fruit flies (Xu et al., 2006) and zebrafish (Khuchua et al., 2006). Subsequent efforts in 2011–2012 led to the development of the first mouse models of BTHS, facilitating tissue-specific studies of tafazzin deficiency in organs such as the heart and skeletal muscle (Acehan et al., 2011; Soustek et al., 2011; Phoon et al., 2012). More recently, BTHS cell models have been developed in immortalized mammalian cell lines, including mouse C2C12 myoblasts (Lou et al., 2018a) and human HEK293 kidney cells, which provide the added experimental benefit of having isogenic control cells.

### Epidemiology

As an X-linked recessive disorder, BTHS is predominantly diagnosed in male patients. It has been suggested that females carrying single-allele TAZ mutations exhibit a skewed pattern of X chromosome inactivation, resulting in a normal clinical presentation (Orstavik et al., 1998). BTHS is exceptionally rare, with only 151 living patients identified worldwide in 2012 (Clarke et al., 2013). Approximately 10 new BTHS cases are diagnosed each year in the United States, which translates to a prevalence of 1:300,000–400,000 live births (Clarke et al., 2013). However, it is likely that BTHS is underdiagnosed due to premature infant mortality and misdiagnosis of children presenting with cardiomyopathies.

### Genetic Basis of BTHS

BTHS results from mutations in the Tafazzin (TAZ) gene (originally referred to as G4.5). TAZ is a mitochondrial transacylase that re-acylates monolysocardiolipin (MLCL) by adding predominantly unsaturated acyl chains (Barth et al., 2004; Schlame and Xu, 2020). It is the primary enzyme that conducts this function, and as such, TAZ mutations result in elevated MLCL levels, decreased overall CL, and increased saturated fatty acid content in CL (Vreken et al., 2000; Schlame et al., 2003; Gu et al., 2004; Valianpour et al., 2005).

TAZ is located on chromosome Xq28 and contains 11 exons (Bolhuis et al., 1991; Bione et al., 1996). Although multiple mRNA splice variants exist, the only detectable form of TAZ protein in human fibroblasts lacks exon 5 (Lu et al., 2016). However, BTHS-associated mutations have been identified in all TAZ exons, including exon 5, suggesting that full-length TAZ protein is also physiologically relevant *in vivo* (Cantlay et al., 1999; Gonzalez, 2005). To date, more than 180 pathogenic TAZ gene mutations have been identified, ranging from single nucleotide polymorphisms to whole-gene deletion (Singh et al., 2009). A major enigma in BTHS research is the apparent discrepancy between specific TAZ mutations and the clinical phenotypes they result in. For example, individuals sharing an identical mutation can have contrasting clinical presentations that range from severe heart failure and hypotonia to being nearly asymptomatic (Ronvelia et al., 2012).

### Pathology

#### Cardiomyopathy

BTHS and its clinical manifestations have been previously discussed in other reviews (Raja et al., 2017b; Ghosh et al., 2019; Taylor et al., 2021; Zegallai and Hatch, 2021). Cardiomyopathy is the major clinical manifestation of BTHS, and all identified patients have developed cardiomyopathy at some point in their lives. Dilated cardiomyopathy is the most common form in BTHS patients and is often associated with hypertrophy, left-ventricular noncompaction, arrhythmia, conduction defects, and endocardial fibroelastosis (a heart disorder in children characterized by diffuse thickening of the endocardium; Brady et al., 2006; Raja et al., 2017b). Hypertrophic and dilated phases can be recurring over a patient’s lifetime (Ferreira et al., 2014). Cardiomyopathy increases the risk of arrhythmia, conduction defects, and congestive heart failure and may lead to sudden cardiac death (Spencer et al., 2006; Yen et al., 2008). In addition, cardiomyopathy in BTHS can be diagnosed late or misdiagnosed, leading to cardiac failure (Spencer et al., 2006; Mangat et al., 2007).

Fortunately, BTHS patient outcomes have improved significantly in the past two decades. Patients born after the year 2000 have a 5-year survival rate of 70% compared to 22% for those born before 2000 (Rigaud et al., 2013). This change suggests that early identification and management of heart dysfunction can significantly improve survival rates for BTHS patients. Although the molecular mechanisms are not known, studies have suggested that several factors, including mitochondrial dysfunction, defective mitochondrial protein import, autophagy, lipid storage myopathy, reduced glucose oxidation, and deficient muscle development, can all contribute to the onset of cardiomyopathy in BTHS (Shen et al., 2015; Greenwell et al., 2021).

#### Skeletal Myopathy

Skeletal myopathy is widely observed in BTHS, and patients often present with a combination of muscle weakness and wasting, delayed gross motor development, pre-pubescent growth delay, and/or hypotonia (Spencer et al., 2006, 2011; Bittel et al., 2018). In BTHS, this condition is usually non-progressive and mainly affects proximal skeletal muscle (Clarke et al., 2013; Ferreira et al., 2014; Mazar et al., 2019) Growth delay usually regresses over time and is often followed by accelerated growth during mid and late puberty (Reynolds et al., 2015). Similar to cardiomyopathy, the mechanism underlying development of skeletal myopathy in BTHS is not well understood.

#### Exercise Intolerance

In a self-assessment, BTHS patients identified exercise intolerance as the clinical feature that most negatively impacts their daily life. The reduced capacity for physical activity in BTHS is thought to result from both cardiac impairment (i.e., overall endurance) and diminished skeletal muscle oxygen utilization (Spencer et al., 2011). Not only does exercise intolerance pose a physical limitation on the ability of patients to independently perform everyday tasks, but it also serves as a psychosocial barrier that likely contributes to reports of lower quality of life ratings from BTHS patients relative to their peers (Storch et al., 2009; Mazar et al., 2019).

#### Neutropenia

The severity of neutropenia in BTHS ranges from mild (benign, transient neutropenia) to severe (congenital neutropenia affecting multiple organs; Folsi et al., 2014). Bacterial or viral infections resulting from neutropenia can cause significant complications throughout a patient’s life, including the risk of death by sepsis in the most extreme cases. According to the first report by Barth et al. (1983) three out of his seven patients died prematurely due to septicemia. The molecular mechanisms underlying the development of neutropenia in BTHS are still largely unknown.

#### Other Clinical Manifestations

While the above pathologies constitute the core clinical features of BTHS, a range of other clinical manifestations have been reported. Some patients develop facial dysmorphism characterized by a tall, broad forehead, round face, full cheeks, and large ears (Ferreira et al., 2014). Additionally, dysmorphism of the feet resulting in talipes equinovarus (clubfoot) has been reported at a higher incidence in BTHS patients (Ades et al., 1993; Spencer et al., 2011). Cognitive and neurological phenotypes have also been described. For example, a study of 15 adolescent BTHS patients found reduced abilities in mathematics and visual spatial tasks (Mazzocco et al., 2007), and a recent study also identified deficiencies in balance and motion reaction time in a group of 33 BTHS patients relative to age-matched controls (Hornby et al., 2019). Other phenotypes include a strong gag reflex (Reynolds et al., 2012), sideways curvature of the spine (Roberts et al., 2012), and increased male fetal loss, stillbirth, and neonatal death (Marziliano et al., 2007; Steward et al., 2010).

## Cardiolipin and Barth Syndrome

### The Cardiolipin Biosynthetic Pathway

#### CL Synthesis

In order to comprehend how the yeast model has furthered our understanding of BTHS pathophysiology, it is necessary to understand the details of the CL biosynthetic pathway, many of which were first discovered in yeast. CL is a dimeric phospholipid in which two phosphatidyl groups are connected by a central glycerol molecule (Lecocq and Ballou, 1964). It is synthesized and localized predominantly in the inner leaflet of the IMM (Hostetler and van den Bosch, 1972; Krebs et al., 1979; Schlame and Haldar, 1993; Gebert et al., 2009; Joshi et al., 2009; Schlame and Ren, 2009; Sparagna and Lesnefsky, 2009; Osman et al., 2011).

**Figure 1:**
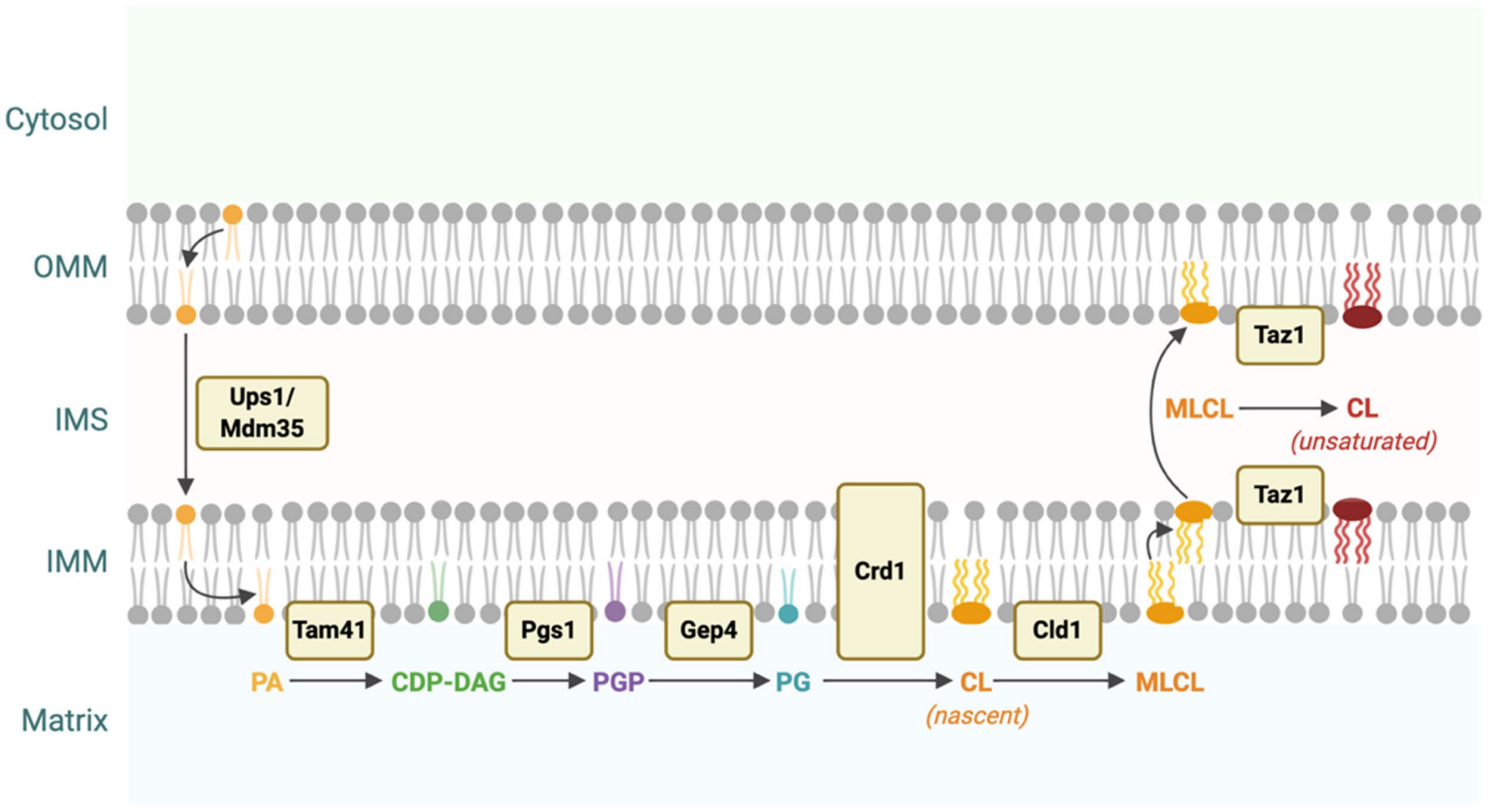
Cardiolipin (CL) biosynthesis in yeast. CL is synthesized from phosphatidic acid (PA) through a four-step process. PA is translocated from the outer mitochondrial membrane (OMM) to the IMM *via* the Ups1/Mdm35 protein complex (Potting et al., 2010; Tamura et al., 2010; Connerth et al., 2012). PA is converted into cytidine diphosphate diacylglycerol (CDP-DAG), phosphatidylglycerol phosphate (PGP), and then phosphatidylglycerol (PG) by Tam41, Pgs1, and Gep4, respectively (Chang et al., 1998a; Osman et al., 2010; Blunsom et al., 2018). Finally, PG is converted to nascent CL by the enzymatic activity of Crd1. Nascent CL, which contains predominantly saturated acyl chains, is remodeled through several cycles of deacylation and reacylation catalyzed by Cld1 and Taz1 to form primarily unsaturated CL (Xu et al., 2006; Beranek et al., 2009). This figure was created with BioRender.com.

CL biosynthesis is a four-step process that utilizes phosphatidic acid (PA) as substrate (Figure 1). PA is synthesized in both the endoplasmic reticulum (ER) and the outer leaflet of the outer mitochondrial membrane (OMM; Chakraborty et al., 1999). In both cases, the translocation of PA from the OMM to the inner leaflet of the IMM *via* the yeast Ups1/Mdm35 lipid transport complex (PRELID1/TRIAP1 in humans) is essential for CL synthesis (Potting et al., 2010; Tamura et al., 2010; Connerth et al., 2012). In the first step of the biosynthetic pathway, PA in the IMM is converted into cytidine diphosphate diacylglycerol (CDP-DAG) by Tam41 in yeast (TAM41 in humans; Blunsom et al., 2018). Next, the yeast mitochondrial enzyme Pgs1 or its human homolog, PGS1, converts CDP-DAG into phosphatidylglycerol phosphate (PGP) by transferring a phosphatidyl group from CDP-DAG onto glycerol-3-phosphate (G3P; Chang et al., 1998a). PGP is then dephosphorylated to phosphatidylglycerol (PG) by the yeast enzyme Gep4 (PTPMT1 in humans; Osman et al., 2010; Zhang et al., 2011). Finally, a phosphatidyl group from CDP-DAG is transferred to PG by the yeast enzyme Crd1 (hCLS1 in humans) to synthesize nascent CL (Schlame and Greenberg, 1997; Chang et al., 1998b).

#### CL Remodeling

Newly synthesized CL undergoes a unique remodeling process in which saturated acyl chains are replaced with unsaturated acyl chains through several cycles of deacylation and reacylation. The yeast enzyme Cld1 is responsible for the first step in CL remodeling by deacylating CL to form MLCL (Beranek et al., 2009). In the second step, MLCL is reacylated by tafazzin (Taz1 in yeast or TAZ in humans) to form primarily unsaturated CL (Figure 1; Xu et al., 2006).

In mammals, multiple enzymes are capable of deacylating CL, though none of them are direct homologs of the yeast enzyme Cld1. These include iPLA2γ, iPLA2β, cPLA2, and sPLA2 (Buckland et al., 1998; Mancuso et al., 2007; Dennis et al., 2011; Hsu et al., 2013). In addition to TAZ, MLCLAT1 and ALCAT1 can also catalyze the reacylation of MLCL in mammalian cells, while there are no direct homologs of these two enzymes in yeast (Ma et al., 1999; Cao et al., 2004; Li et al., 2010; Mejia et al., 2018). As a result of diminished tafazzin function, BTHS is characterized by an increase in MLCL levels, along with a decrease in unsaturated and total CL (Jiang et al., 1997; Xu et al., 2006; Lou et al., 2018a; Wang et al., 2020). The fact that these phenotypes arise despite the presence of MLCLAT1 and ALCAT1 suggests that TAZ is the primary enzyme responsible for reacylation of MLCL under physiological conditions (Saric et al., 2015).

## Studying Cl-Related Pathologies Using Yeast Mutants

Yeast is an excellent model for studying genetic disorders. In 1996, *S. cerevisiae* was the first eukaryote for which a full genome sequence was assembled (Goffeau et al., 1996; Giaever and Nislow, 2014). Subsequently, researchers generated a deletion collection containing representative mutant strains for each nonessential gene in the yeast genome (~4,800 genes; Giaever and Nislow, 2014). Using these tools, homologs of over 23% of all human genes have been identified and studied in yeast (Kachroo et al., 2015). One of the most striking discoveries from these endeavors was that many genes encoding lipid pathways are conserved from yeast to humans, thus making yeast a useful model to interrogate human diseases of lipid metabolism. The ability of yeast to grow both as haploid or diploid cells allows for the construction of double mutants by crossing single mutants, inducing sporulation, and screening the resulting haploids. In addition to being genetically tractable, yeast cells are nonpathogenic, have a short generation time, and are easy and inexpensive to culture in the laboratory.

The CL biosynthetic pathway is conserved from yeast to humans, and yeast mutants have been constructed for each step of this pathway. Among these, the *crd1*Δ mutant (Jiang et al., 1997; Tuller et al., 1998; Chang et al., 1998b), which lacks CL synthase and cannot synthesize CL, and the *taz1*Δ mutant (Vaz et al., 2003; Gu et al., 2004; Ma et al., 2004), lacking tafazzin, have been pivotal in elucidating the cellular roles of CL and understanding BTHS pathology. Yeast mutants have also been generated to recapitulate and test the functional significance of human TAZ mutations identified in BTHS patients (Claypool et al., 2006, 2011). The following sections detail pioneering discoveries relevant to BTHS pathophysiology that were first made using yeast mutants.

### CL and Bioenergetics

CL comprises 15–20% of the phospholipid content in the IMM where it plays a pivotal role in energy metabolism (Pennington et al., 2019). The mitochondrial III_2_IV_2_ supercomplex forms the terminal part of the electron transport chain and is essential for maintaining mitochondrial membrane potential and ATP synthesis (Schagger and Pfeiffer, 2000). In *crd1*Δ yeast, the III_2_IV_2_ supercomplex is less stable than in wildtype cells, suggesting that CL is critical for mitochondrial homeostasis and bioenergetics (Pfeiffer et al., 2003; Mileykovskaya et al., 2005; Zhang et al., 2005; Claypool et al., 2008; Bottinger et al., 2012; Bazan et al., 2013; Peyta et al., 2016; Petit et al., 2020). A deficiency in CL remodeling is also associated with reduced bioenergetics (Brandner et al., 2005; Li et al., 2007). Using *taz1*Δ yeast, Brandner et al. (2005) demonstrated that the absence of tafazzin results in increased dissociation of the III_2_IV_2_ supercomplex, causing the release of a complex IV monomer (Brandner et al., 2005; Claypool et al., 2008).

The initial yeast studies implicating CL and CL remodeling in bioenergetics were subsequently corroborated using mammalian BTHS models. Gonzalvez et al. (2013) and McKenzie et al. (2006) reported decreased respiratory supercomplex formation and stability in lymphoblast cells isolated from two BTHS patients relative to non-BTHS control cells. More recently, Petit et al. (2020) showed that shRNA-mediated Taz knockdown in HeLa cells results in decreased ATP synthase activity and overall ATP level, with a concomitant decrease in maximal respiratory capacity and an increase in basal oxygen consumption. Similarly, Dudek et al. (2013, 2016) showed reduced respiratory complex formation in cardiac tissue isolated from the BTHS mouse model and increased basal oxygen consumption coupled with decreased maximal respiratory capacity in induced pluripotent stem cells (iPSCs) derived from BTHS patients. Ma et al. (2004) reported a decrease in basal respiration in *taz1*∆ yeast, a study corroborated by Lou et al. (2018a) in C2C12 TAZ KO (Ma et al., 2004). The increased basal respiration observed in iPSC-derived cardiomyocytes could be due to increased F1F0 ATP synthase oxygen consumption and proton leak. The authors further showed that the aberrant respiration in iPSCs could be attributed to reduced respiratory complex stability, reiterating what was previously concluded from yeast. These findings underscore the power of the yeast model for interrogating the relationship between CL deficiency and the bioenergetic defects that characterize BTHS.

### CL and Iron Homeostasis

Iron–sulfur clusters (ISCs) are molecular assemblages of iron and sulfur atoms that act as co-factors in many cellular processes, including electron transfer within the electron transport chain and enzymatic conversion of substrate within the TCA cycle (Paul et al., 2017; Braymer et al., 2021). ISCs can exist in many configurations based on the number of iron and sulfur atoms involved (e.g., 2Fe-2S, 3Fe-4S, and 4Fe-4S). ISC biogenesis occurs in three steps within mitochondria (Lill and Freibert, 2020). The first step involves donation of sulfur from the NFS1-ISD11-ACP1 subcomplex and transfer of imported iron to the ISCU2 scaffold protein by frataxin (FXN). This forms an initial 2Fe-2S cluster. In the second step, chaperone proteins (HSC20, HSPA9, and GRPE1) bind to the 2Fe-2S cluster and transfer it first to the monothiol glutaredoxin GLRX5 and subsequently to mitochondrial ISC recipient proteins. The third step involves conversion of 2Fe-2S clusters into 4Fe-4S clusters and their delivery to recipient apoproteins (Lill and Freibert, 2020; Maio et al., 2020).

The first indication of a relationship between CL and ISC homeostasis was described in yeast. Using *crd1*Δ yeast, Patil et al. (2013) demonstrated that CL deficiency results in elevated mitochondrial iron and increased sensitivity to ROS and exogenously supplied iron sulfate. These phenotypes are associated with defective ISC biogenesis, and the authors subsequently showed that *crd1*Δ cells exhibit decreased enzymatic activity of the ISC-requiring enzymes ubiquinol-cytochrome *c* oxidoreductase, succinate dehydrogenase, aconitase, isopropylmalate isomerase, and sulfite reductase. Additionally, deletion of the ISC biosynthetic gene ISU1 in the *crd1*Δ background resulted in a synthetically sick phenotype, further supporting a role for CL in iron homeostasis (Patil et al., 2013).

These initial findings from yeast were subsequently validated in mammalian cells using the TAZ-KO C2C12 BTHS cell model. Similar to what was observed in yeast, Li et al. (2020) demonstrated that the activities of the ISC-requiring enzymes aconitase, NADH dehydrogenase, succinate dehydrogenase, and ubiquinol-cytochrome *c* reductase were all decreased by ~50% in TAZ-KO cells while their respective protein levels remained unchanged. TAZ-KO cells also showed increased mitochondrial iron content and elevated sensitivity to ROS and iron supplementation, mirroring the findings from yeast. This study went on to further corroborate the role of CL in ISC biogenesis by showing that the mature form of the ISC biosynthetic protein FXN is reduced in TAZ-KO cells (Li et al., 2020).

### CL and Energy Metabolism

Raja et al. (2017a) provided the first evidence of a link between CL and energy metabolism by showing that *crd1*Δ exhibits decreased synthesis of acetyl-CoA, and that deletion of *CRD1* is synthetically lethal in pyruvate dehydrogenase (PDH) mutants. Acetyl-CoA is a primary substrate utilized by the tricarboxylic acid (TCA) cycle to fuel intermediary metabolism, and under respiratory conditions it is synthesized predominantly in mitochondria through the enzymatic conversion of pyruvate by PDH (Guest et al., 1989; Raja et al., 2017a). Synthetic lethality between *crd1*Δ and PDH complex mutants suggests that CL plays a role in promoting acetyl-CoA synthesis and TCA cycle function. Interestingly, PDH complex mRNA and protein levels are increased in *crd1*Δ, but net activity of PDH is not altered (Raja et al., 2017a). This suggests that CL is required for optimal PDH function, and that in the absence of CL, upregulation of PDH cannot compensate for diminished acetyl-CoA synthesis.

Building on the findings in yeast, Li et al. (2019) found that TAZ-KO mouse C2C12 cells also show reduced carbon flux to acetyl-CoA, and this is linked to a reduction in PDH activity. PDH is regulated through phosphorylation, and the authors showed that both the inhibitory phosphorylation and enzymatic activity in mitochondrial extracts are rescued by the addition of exogenous CL. Although production of acetyl-CoA through the activity of PDH serves as an important input for the TCA cycle, the cycle itself is comprised of many steps that are each catalyzed by distinct enzymes, some of which require Fe-S cofactors for optimal activity. Li et al. (2019, 2020) identified a second way in which CL deficiency negatively impacts TCA cycle function by showing that activity of the Fe-S-requiring TCA cycle enzymes succinate dehydrogenase and aconitase are also reduced in TAZ-KO cells.

### CL Enhances the Stability of the Mitochondrial Calcium Uniporter

The mitochondrial calcium uniporter (MCU) is a holo-complex protein consisting of pore-forming subunit MCU, transmembrane subunit EMRE, and regulatory subunits MICU1, MICU2, and MCUb (Baughman et al., 2011; De Stefani et al., 2011; Sancak et al., 2013). MCU transports calcium from the cytosol into the mitochondria where it serves as a signal for regulating ATP synthesis and, when in excess, activating the apoptotic pathway (Carraro et al., 2020). MCU is localized in the IMM where CL is enriched, suggesting that its function could be influenced by CL (Ghosh et al., 2020).

The first evidence for CL playing a role in MCU function came from a recent study in yeast, where Ghosh et al. (2020) reported that MCU levels are decreased by 50% in CL-deficient *crd1*Δ cells. In this study, MCU was heterologously expressed under a strong promoter. Therefore, the authors argue that CL influences MCU stability but not expression, which is supported by the finding that the MCU turnover rate is substantially higher in *crd1*Δ compared to wildtype cells. As a functional consequence, mitochondrial uptake of calcium is also decreased in *crd1*Δ mutants. The authors further validated these yeast findings in both the TAZ-KO C2C12 mouse model and in BTHS patient-derived lymphoblasts and heart tissue. In a similar study, Ghosh et al. (2021) showed that only the MICU1 component of the mitochondrial calcium uniporter complex is decreased in *crd1*Δ cells. In this study, human MICU1 and Mitochondrial Calcium Uniporter Regulator 1 (MCUR1) proteins were expressed in both wildtype and *crd1*Δ yeast cells. The authors showed that MICU1 levels were 50% lower in *crd1*Δ cells compared to the wildtype, while MCUR1 levels were unaltered (Ghosh et al., 2021). This work serves as yet another example of how yeast research has led to pioneering discoveries regarding CL function and its relationship to BTHS.

## Using Yeast To Study Other Lipid-Related Diseases

The yeast model has proven to be indispensable not only in the study of CL-related pathologies, but in other lipid-related pathologies as well. In addition, the genetic tractability, rapid doubling time, and low cost often make the use of yeast preferable to other models. When modeling human diseases in yeast there are two general approaches. The first method is referred to as an orthologous approach, in which a yeast ortholog of a human gene is modified to have the same mutation as in the human disease (e.g., the *crd1*Δ and *taz1*Δ strains used to study BTHS). Using this approach researchers can identify interactions between other genes, proteins, and molecules that may interact with the aberrant disease pathway. However, this is not always possible, as the discrepancy in genome size between yeast and humans means that not all human genes have a yeast homolog. The second method, referred to as humanization, involves expressing a human disease gene in yeast. Although some tissue-wide aspects of human diseases are difficult to model in the unicellular yeast system, cellular phenotypes can often be recapitulated and studied in yeast cells. The following sections detail ways in which yeast have been used to study the lipid-related pathophysiology of two prevalent human neurodegenerative diseases, Alzheimer’s and Parkinson’s.

### Alzheimer’s Disease

Alzheimer’s disease (AD) is the most common neurodegenerative disease in the world and is characterized by the extracellular accumulation of amyloid-β (Ab) peptide in senile plaques and the intracellular accumulation of neurofibrillary tangles (Masters et al., 1985; Alzheimer et al., 1995; Scheltens et al., 2016). These aggregations lead to neurodegeneration, with the main clinical manifestation being severe memory impairment and memory loss that often results in chronic dementia (Masliah et al., 1989; Bancher et al., 1997; Scheltens et al., 2016). While the molecular phenotypes of AD have been well cataloged, the exact pathophysiology of the disease is not well understood.

In order to gain insight into the pathophysiology of AD, Nair et al. (2014) conducted a genome-wide screen to identify cellular processes that influence Ab aggregation in yeast. In this study, the authors compared mutants from the yeast deletion collection expressing a GFP-tagged Ab construct (Ab-GFP) with wild-type cells to identify genes that influence the size or localization of Ab aggregations. Out of ~4,600 mutants tested, the screen identified 110 relevant genes corresponding predominantly to four major cellular processes, including phospholipid metabolism, gene expression, chromatin remodeling, and mitochondrial function. This study has been foundational in guiding subsequent AD research efforts.

In particular, dysregulation of lipid metabolism has been increasingly recognized as a contributing factor to AD pathophysiology, and the yeast model has been indispensable for exploring this link (Kao et al., 2020). One example of the link between lipid metabolism and AD pathology involves a neurotoxic species of phosphatidylcholine, referred to as PC(*O*-16:0/2:0), that is elevated in human AD tissue (Ryan et al., 2009). Kennedy et al. (2016) used yeast to better understand the role of this lipid species in AD pathology by combining gene expression profiling with a genome-wide chemogenomic screen. They found that elevated PC(*O*-16:0/2:0) causes an accumulation of ceramide that ultimately results in increased reactive oxygen species (ROS) production and mitochondrial dysfunction, cellular phenotypes that are commonly seen in AD patient cells and AD cell models (Agrawal and Jha, 2020).

Another example of the link between lipid metabolism and AD relates to tau protein phosphorylation. Tau hyperphosphorylation has been implicated as a major part of AD neurodegeneration, but the mechanism of hyperphosphorylation is not well understood (Simic et al., 2016). Using yeast, Randez-Gil et al. (2020) identified a potential mechanism wherein dysregulation of inositol phosphate signaling leads to defective sphingolipid production and a resultant increase in tau protein hyperphosphorylation. Although sphingolipid metabolism had been previously linked to neurodegenerative diseases (Alaamery et al., 2021), this was one of the first studies to suggest a role for SLs in tau hyperphosphorylation.

The previous studies directly link lipid metabolism to AD, but other aspects of lipid homeostasis have also been shown to contribute to AD pathology. Apolipoprotein E (APOE) is a lipid transporter that transports lipids between cells and tissues (Huang and Mahley, 2014). APOE is polyallelic, and the *E4* allele (APOE4) is a known risk factor for AD (Farrer et al., 1997). It is well characterized that APOE4 plays a role in AD, but the mechanism by which it contributes to AD is largely unknown. Using genome-wide screens and lipidomic analysis in yeast, Sienski et al. (2021) determined that APOE4 is responsible for altered fatty acid (FA) metabolism which is commonly seen in AD cells. They found that when human APOE4 is expressed in yeast there is an increase in the degree of unsaturation in FAs accompanied by the accumulation of lipid droplets. Furthermore, they also demonstrated that choline supplementation ameliorates the aberrant FA metabolism (Sienski et al., 2021). When choline was supplemented, synthesis of the membrane phospholipid phosphatidylcholine was stimulated. This abolished APOE4 lipid-related defects and suggests an important role for phosphatidylcholine in the pathophysiology of AD. This study demonstrated not only a novel role of APOE4 in AD, but also identified a key modulator of this process, choline homeostasis.

Yeast has proved to be a powerful tool for studying lipid-related pathology in AD. By combining the genetic tractability of yeast with high-throughput screening techniques, researchers have uncovered novel mechanisms into the influence of lipid homeostasis on Ab aggregation, ROS production, tau hyperphosphorylation, and FA metabolism.

### Parkinson’s Disease

Parkinson’s disease (PD) is the second most common neurodegenerative disease world-wide. PD is characterized by degeneration of nigrostriatal dopaminergic neurons in the substantia nigra pars compacta region of the brain and the presence of intraneuronal α-synuclein (aS) inclusions known as Lewy bodies (Antony et al., 2013; Leao et al., 2015; Sveinbjornsdottir, 2016). Degradation of dopaminergic neurons in this region of the brain decreases dopamine release in the striatum, altering motor control in afflicted individuals (Antony et al., 2013; Leao et al., 2015; Sveinbjornsdottir, 2016). Common motor symptoms include bradykinesia, rigidity, tremors, and postural instability, and these presentations are often accompanied by depression, anosmia, dementia, and sleep disorders (Moustafa et al., 2016; Pfeiffer, 2016). The molecular hallmarks of PD are well characterized, but just as in AD, the exact cause of PD is not known.

One of the major molecular hallmarks of PD is the aggregation of aS, a protein commonly expressed in neurons and enriched in presynaptic terminals (Maroteaux et al., 1988; Taguchi et al., 2016). It is well known that aS aggregation has a central role in PD pathogenesis, but the mechanism by which aS aggregates form is not known. Wang et al. (2014) used the yeast model to test a potential mechanism for aS aggregation and found that phosphatidylethanolamine (PE) deficiency in yeast cells causes ER stress, vesicle defects, aS aggregation, and cells death. Subsequently, the authors demonstrated that the effects of PE deficiency can be mitigated by ethanolamine supplementation (Wang et al., 2014). This study highlights the importance of lipid homeostasis on aS aggregation.

A study by Fanning et al. (2019) sought to further interrogate the relationship between aS aggregation, lipid homeostasis, and cellular toxicity. The authors performed lipidomic profiling in yeast displaying aS aggregation to monitor changes in specific lipid classes. They determined that dysfunctional lipid homeostasis, induced by aS aggregation, leads to cytotoxicity due to the accumulation of oleic acid (OA) and diglycerides (DG) in lipid droplets of aS-expressing yeast (Fanning et al., 2019). Of particular interest from a therapeutic standpoint, they found that either preventing the conversion of triglycerides (TG) to DG or inhibiting stearoyl-CoA desaturase (SCD; the rate-limiting enzyme in the production of OA) ameliorates aS aggregation and its associated cytotoxicity.

Yeast is not only an excellent model for identifying novel pathophysiological mechanisms but also for probing and testing chemical and genetic modifiers of a disease (Griffioen et al., 2006; Williams et al., 2007; Su et al., 2010). For example, Soste et al. (2019) conducted a screen for genetic modifiers of aS aggregation and identified 33 genes that modulate aS aggregation and cytotoxicity. One of these modifiers, Pah1, is an enzyme that converts PA to DG in yeast. In accordance with the findings of Fanning et al. (2019), Soste et al. (2019) found that decreasing DG levels through the inhibition of Pah1 ameliorates aS aggregation and cytotoxicity. This suggests a key role for DG and lipid droplet homeostasis in aS aggregation and cytotoxicity in PD.

Collectively, these studies demonstrate the power of the yeast system for investigating the lipid-related pathophysiology of two prominent neurodegenerative disorders. The unicellular nature of yeast makes it particularly straightforward to track biochemical changes in molecules such as lipids.

## Conclusion

This review outlines some of the major contributions the yeast model has made to our understanding of the lipid-related pathologies observed in BTHS, AD, and PD. Lipid homeostasis has been shown to play key roles in other diseases, including cancer (Snaebjornsson et al., 2020), heart disease (Poss et al., 2020; Richardson et al., 2020), diabetes mellitus (Katsiki et al., 2017; Bjornstad and Eckel, 2018), and other chronic conditions (Leuti et al., 2020). The multifaceted roles of lipids in human disease are only beginning to be uncovered, and as technologies such as mass spectrometry continue to evolve, the yeast model system promises to continue facilitating progress in this rapidly growing field (Zullig and Kofeler, 2021).

## Author Contributions

TR-E, CO, MS, LV, and MG all contributed to the conception, design, and writing of the manuscript. AL contributed to the writing of the manuscript. All authors contributed to the article and approved the submitted version.

## Funding

The Greenberg lab gratefully acknowledges support from grants R01 HL 117880, R01 GM 134715, and RO1 GM125082 from the National Institutes of Health.

## Conflict of Interest

The authors declare that the research was conducted in the absence of any commercial or financial relationships that could be construed as a potential conflict of interest.

## Publisher’s Note

All claims expressed in this article are solely those of the authors and do not necessarily represent those of their affiliated organizations, or those of the publisher, the editors and the reviewers. Any product that may be evaluated in this article, or claim that may be made by its manufacturer, is not guaranteed or endorsed by the publisher.
